# Relationship between the microbiome and obesity-associated cancer risk using Mendelian randomisation

**DOI:** 10.1038/s41366-026-02032-3

**Published:** 2026-02-19

**Authors:** Thomas Yates, Molly Went, Charlie Mills, Philip Law, Richard Houlston

**Affiliations:** Division of Genetics and Epidemiology, The Institute of Cancer Research, Sutton, Surrey UK

**Keywords:** Cancer, Genetics

## Abstract

**Background:**

The mechanisms underlying obesity-related cancer risk are incompletely understood. We investigated whether the gut microbiome causally mediates this relationship.

**Methods:**

We performed two-sample Mendelian randomisation, with mediation analysis, to assess causal links between genetically predicted body mass index (BMI)/waist-to-hip ratio adjusted for BMI (WHRadjBMI), 211 gut microbial taxa, and eight cancers (384,738 cases) of European ancestry. Significant associations were replicated in the FinnGen cohort.

**Results:**

Genetically predicted BMI was associated with risk of colorectal (CRC; odds ratio per standard deviation (OR_SD_): 1.12; 95% confidence interval (CI): [1.06–1.17]; *P* = 4.95 × 10^−6^), kidney (RCC) (OR_SD_: 1.48; 95% CI: [1.34–1.63]; *P* = 1.61 × 10^−15^), endometrial (OR_SD_: 1.70; 95% CI: [1.55–1.87]; *P* = 2.09 × 10^−27^), lung (OR_SD_: 1.20; 95% CI: [1.12–1.29]; *P* = 1.40 × 10^−7^), and oesophageal cancer (OR_SD_: 1.25; 95% CI: [1.13–1.39]; *P* = 3.09 × 10^−5^). Seven microbial taxa were associated with CRC risk. Phylum and class Actinobacteria showed the strongest effects (OR_SD_: 1.48; 95% CI: [1.29–1.70]; *P* = 1.78 × 10^−8^) and (OR_SD_: 1.36; 95% CI: [1.22–1.51]; *P* = 2.57 × 10^−8^), respectively, and replicated in FinnGen, mediating 29% (95% CI: [8-50]) and 21% (95% CI: [4–37]) of the BMI to CRC risk—collectively accounting for 50% of the relationship. No consistent microbiome mediation was observed for other cancers.

**Conclusions:**

Gut Actinobacteria may contribute to obesity-driven CRC risk, supporting the rationale of microbiome-targeted interventions to reduce CRC risk.

## Introduction

Obesity is a major global health challenge and an established risk factor for several chronic diseases [1]. In addition to its well-recognised role in cardiovascular disease, obesity contributes to the development of several malignancies including colorectal (CRC), renal cell (RCC), endometrial, oesophageal, and pancreatic cancers [2]. Although metabolic, hormonal, and inflammatory mechanisms have been implicated, the biological pathways linking obesity to cancer risk remain incompletely understood.

Emerging evidence suggests that the gut microbiome may play a role in mediating the effects of obesity on carcinogenesis [3, 4]. However, establishing a causal relationship between the microbiome and cancer using conventional observational studies has proven difficult due to residual confounding and reverse causality, particularly from lifestyle factors that influence both microbial composition and cancer risk [5].

**Fig. 1:**
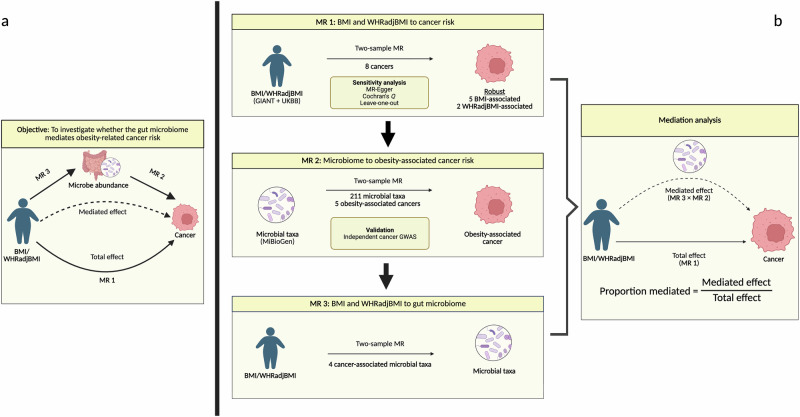
Study design. **a** Study overview. **b** The individual stages of the study. Arrows indicate that only significant associations from previous MR analyses are carried forward. Mediation analysis is therefore only performed for trios of BMI/WHRadjBMI, microbial taxa, and cancer for which they are all significantly associated. MR Mendelian randomisation, BMI body mass index, WHRadjBMI waist-to-hip ratio adjusted for BMI, UKBB UK Biobank.

To address these limitations, we employed Mendelian randomisation (MR), an analytical approach that uses genetic variants as instrumental variables (IVs) to infer causal relationships with reduced susceptibility to confounding and reverse causation [6, 7] (Fig. 1). Specifically, we conducted two-sample MR (2S-MR) and formal mediation analyses to investigate whether genetically predicted obesity increases cancer risk through gut microbial dysbiosis. Using the largest available genome-wide association study (GWAS) datasets for obesity-related traits, the gut microbiome, and cancer consortia, we examined potential causal relationships between obesity, the microbiome, and eight cancers—breast, prostate, CRC, lung, endometrial, oesophageal, RCC, and ovarian cancer—comprising a total of 384,738 cases and 799,908 controls of European ancestry.

## Materials and methods

We first performed 2S-MR to test for genetically predicted associations between obesity and cancer risk. Here, body mass index (BMI) and waist-hip ratio adjusted for BMI (WHRadjBMI) were used as measures of obesity (Table S1). Genetic variants associated with BMI/WHRadjBMI were identified using GWAS meta-analysis data of individuals with European ancestry from the GIANT consortium and the UK Biobank (BMI, 681,275 samples; WHRadjBMI, 694,649 samples) [8, 9]. We used summary cancer GWAS effect estimates from: (1) Online consortia resources, for breast (BCAC; https://bcac.ccge.medschl.cam.ac.uk/, accessed July 2022) and prostate cancer (PRACTICAL; http://practical.icr.ac.uk/; accessed July 2022); (2) GWAS Catalogue (https://www.ebi.ac.uk/gwas/), for ovarian, CRC, endometrial, and lung cancers (accessed September 2022); (3) Investigators of published work, for RCC and oesophageal cancer. Since the UK Biobank was used to obtain genetic instruments for obesity traits, the CRC and oesophageal GWAS association statistics were recalculated from primary data excluding UK Biobank samples to avoid sample overlap bias (Table S2).

Single nucleotide polymorphisms (SNPs) acting as instrumental variables (IVs) for the exposures were identified from GWAS summary statistics using PLINK v.1.9 [10, 11]. All were independently genome-wide significant (*i.e*., *P* < 5 × 10^−8^; r^2^ < 0.01, within a clumping window of 500 kb) and had a minor allele frequency > 0.01, referenced to the 1000 Genomes Project European panel (Tables S3 and S4). SNPs within the human leukocyte antigen complex were excluded, due to potential horizontal pleiotropy, and the IV for each linkage disequilibrium (LD) block was chosen as the SNP with the lowest *P*-value.

Data harmonisation and MR analyses were conducted using TwoSampleMR v.0.5.9 [12, 13], with SNPs not present in both GWAS removed. Where multiple IVs were available, the inverse variance weighted random-effects (IVW-RE) method [14] was used to estimate the effect size, and the MR-Egger intercept test [15], Cochran’s *Q* statistics, and leave-one-out analysis [16] were used to ensure robustness of any associations. The Wald-ratio [17] was used for exposures with only a single IV. We adjusted for the number of cancers by using a Bonferroni-corrected threshold of *P* < 6.25 × 10^−3^ (0.05/8). The level of statistical significance was categorised as either robust (*P* < 6.25 × 10^−3^) or nominal (6.25 × 10^−3^ < *P* < 0.05). Robust associations were taken forward for mediation analysis. *F*-statistics [18] and power calculations [19] were used to test for potentially false null associations and bidirectional MR analysis was used to test for the correct causal direction.

We then used 2S-MR to test for potential associations between the gut microbiome and obesity-driven cancer risk. For the microbiome, we used published summary GWAS effect estimates of 211 microbial taxa in 18,340 individuals from the MiBioGen consortium [20], with IVs defined as in the previous step (Table S5). Whilst a small clumping window size maximises the number of instruments and power, it increases the risk of using correlated instruments, violating the MR independence assumption. We therefore validated significant microbe-cancer associations using instruments clumped adopting a 1 Mb window. A Bonferroni-corrected *P*-value threshold for each taxonomic rank per cancer type was used to define statistical significance. Significant associations were tested for replication in the FinnGen cohort [21] and for association with colon and rectal cancers.

The effect of obesity on cancer-associated microbes was then tested using 2S-MR. We used the same IVs as in the first step and the same sensitivity analyses were used to test significant associations.

Microbes found to be associated with both obesity and cancer risk were then tested for mediation of obesity-driven cancer risk. Due to the scarcity of IVs for the microbiome, mediation analysis was undertaken using the product method [22]. In the product method, the effect estimate of obesity on the microbe and the effect estimate of the microbe on cancer risk are multiplied to calculate the mediated effect estimate. The standard error of the mediated effect is calculated using the delta method. The proportion of the total effect of obesity on cancer risk mediated through the microbe can be estimated as the ratio of the microbe-mediated effect and the total effect of obesity on cancer risk. To avoid pleiotropy, SNPs that were IVs for both the obesity measure and the microbe (or within 500 kb of a microbe IV) were not used to calculate obesity measure effect estimates [23].

Evidence from the World Cancer Research Fund and the American Institute for Cancer Research links alcohol consumption, physical activity, and dietary factors to cancer risk [24]. Thus, IVs for potential microbe mediators were manually reviewed for pleiotropic associations using data from the GWAS Catalogue [25].

## Results

For all eight cancer types, the *F*-statistics were greater than 55.8 for BMI and 54.2 for WHRadjBMI, hence there was no evidence of weak instrument bias (i.e., *F*-statistic < 10) [26]. Power calculations for odds ratios (OR_SD_) per standard deviation (S.D.) between 1.05 and 1.50 are given in Tables S6 and S7.

Genetically predicted obesity was associated with an increased risk of several cancers (Table 1). Specifically, a per S.D. increase in genetically predicted BMI showed robust associations with increased risk of CRC (OR_SD_: 1.12; 95% confidence interval (CI): [1.06, 1.17]), RCC (OR_SD_: 1.48; 95% CI: [1.34, 1.63]), endometrial cancer (OR_SD_: 1.70; 95% CI: [1.55, 1.87]), lung cancer (OR_SD_: 1.20; 95% CI: [1.12, 1.29]), and oesophageal cancer (OR_SD_: 1.25; 95% CI: [1.13, 1.39]), and nominal associations with increased risk of breast (OR_SD_: 0.94; 95% CI: [0.89, 0.98]) and ovarian (OR_SD_: 1.07; 95% CI: [1.00, 1.15]) cancer. A per S.D. increase in genetically predicted WHRadjBMI also showed robust associations with an increased risk of CRC (OR_SD_: 1.14; 95% CI: [1.07, 1.21]) and oesophageal cancer (OR_SD_: 1.24; 95% CI: [1.07, 1.43]), and a nominal association with an increased risk of RCC (OR_SD_: 1.16; 95% CI: [1.03, 1.31]); however, there was no significant association between WHRadjBMI and endometrial, lung, breast, or ovarian cancer. No reverse causation was detected using bidirectional MR (Table S8). Obesity was not shown to be associated with risk of prostate cancer.

**Table 1:** MR results for associations between BMI/WHRadjBMI and cancer risk.

Cancer	Obesity measure	N_SNP	OR_SD	95% CI	P-value	Pleiotropy	Heterogeneity	Driven by single SNP
CRC	BMI	1064	1.12	[1.06, 1.17]	4.95 × 10 − 6	FALSE	FALSE	FALSE
	WHRadjBMI	635	1.14	[1.07, 1.21]	2.51 × 10 − 5	FALSE	FALSE	FALSE
RCC	BMI	1067	1.48	[1.34, 1.63]	1.61 × 10 − 15	FALSE	FALSE	FALSE
	WHRadjBMI	622	1.16	[1.03, 1.31]	1.27 × 10 − 2	FALSE	FALSE	FALSE
Endometrial	BMI	1064	1.70	[1.55, 1.87]	2.09 × 10 − 27	FALSE	FALSE	FALSE
	WHRadjBMI	630	0.94	[0.83, 1.05]	0.28	FALSE	FALSE	FALSE
Lung	BMI	999	1.20	[1.12, 1.29]	1.40 × 10 − 7	FALSE	FALSE	FALSE
	WHRadjBMI	587	0.93	[0.85, 1.01]	8.26 × 10 − 2	FALSE	FALSE	FALSE
Oesophageal	BMI	910	1.25	[1.13, 1.39]	3.09 × 10 − 5	FALSE	FALSE	FALSE
	WHRadjBMI	499	1.24	[1.07, 1.43]	3.70 × 10 − 3	FALSE	FALSE	FALSE
Breast	BMI	939	0.94	[0.89, 0.98]	7.56 × 10 − 3	FALSE	FALSE	FALSE
	WHRadjBMI	560	0.96	[0.9, 1.02]	0.17	FALSE	FALSE	FALSE
Ovarian	BMI	1038	1.07	[1.00, 1.15]	4.73 × 10 − 2	FALSE	FALSE	TRUE
	WHRadjBMI	619	0.99	[0.9, 1.07]	0.73	FALSE	FALSE	FALSE
Prostate	BMI	762	0.94	[0.88, 1.01]	9.76 × 10 − 2	FALSE	FALSE	FALSE
	WHRadjBMI	460	1.02	[0.93, 1.13]	0.64	FALSE	FALSE	FALSE

Pleiotropy was detected using the MR-Egger intercept (i.e., P_Egger-intercept < 0.05). Heterogeneity was detected using Cochran’s Q statistics (i.e., *P* < 0.05). The association was considered to be driven by a single SNP if the association was no longer nominally significant (i.e., *P* > 0.05) when that SNP was removed from the analysis.

*CRC* colorectal cancer, *RCC* renal cell carcinoma, *BMI* body mass index, *WHR* waist-hip ratio, *OR_SD* odds ratio per standard deviation, *CI* confidence interval, *N_SNP* number of IVs used in the MR analysis.

Focusing on the five robust obesity-cancer associations, we explored whether there was a relationship between cancer risk and the gut microbiome (Table S9). For all microbe-cancer pairs, the *F*-statistics were greater than 29.3, hence there was no evidence of weak instrument bias. Of the microbe-cancer pairs, 64% had sufficient power ( > 0.8) to detect a relationship, provided OR_SD_ was >1.50 (Table S10). A per S.D. increase in the genetically predicted abundance of phylum (OR_SD_: 1.48; 95% CI: [1.29, 1.70]) and class (OR_SD_: 1.36; 95% CI: [1.22, 1.51]) Actinobacteria, order Bifidobacteriales (OR_SD_: 1.28; 95% CI: [1.06, 1.53]), family Bifidobacteriaceae (OR_SD_: 1.28; 95% CI: [1.06, 1.53]), and genus Tyzzerella3 (OR_SD_: 1.30; 95% CI: [1.14, 1.48]), as well as a per S.D. decrease in family Oxalobacteraceae (OR_SD_: 0.80; 95% CI: [0.69, 0.91]) and genus Ruminococcus torques group (OR_SD_: 0.58; 95% CI: [0.46, 0.74]), were associated with increased CRC risk. Although both order Bifidobacteriales and family Bifidobacteriaceae showed evidence of a single SNP driving the associations with CRC risk, the MR-Egger [15] and weighted median effect [27] estimates showed consistent direction of effect with the IVW-RE effect estimates. Therefore, these taxa were not excluded from further analyses. No significant reverse associations were detected (Table S11).

The associations of phylum/class Actinobacteria, order Bifidobacteriales, and family Bifidobacteriaceae remained significant using instruments clumped with a window size of 1 Mb (Table S12) and were replicated in the FinnGen cohort (Table S13). To further investigate potential associations of gut microbes with CRC risk, we tested for significant associations between the CRC-associated microbes and colon and rectal cancers (Tables S14 and S15). All Actinobacteria and Bifidobacteria taxa were robustly associated with both colon and rectal cancers.

We investigated whether the CRC-associated microbes were potential mediators of obesity-driven CRC risk. Correcting for the number of CRC-associated microbes, we imposed a Bonferroni-corrected threshold of *P* < 1.25 × 10^−2^ (i.e., 0.05/4). MR analysis provided evidence of a significant association between BMI and both Actinobacteria taxa (Table 2); however, no significant associations were detected between WHRadjBMI and the cancer-associated microbes. Bidirectional MR analysis was not performed due to a lack of instrumental variables for the microbes after harmonisation.

**Table 2:** MR results for associations between BMI/WHRadjBMI and cancer-associated microbes.

Microbe	Obesity measure	N_SNP	OR_SD	95% CI	*P*-value	Pleiotropy	Heterogeneity	Driven by single SNP
Class Actinobacteria	BMI	1035	1.07	[1.02, 1.13]	9.33 × 10 − 3	FALSE	FALSE	FALSE
	WHRadjBMI	565	1.04	[0.98, 1.12]	0.22	FALSE	FALSE	FALSE
Phylum Actinobacteria	BMI	1035	1.08	[1.03, 1.14]	3.59 × 10 − 3	FALSE	FALSE	FALSE
	WHRadjBMI	565	1.04	[0.98, 1.1]	0.22	FALSE	FALSE	FALSE
Order Bifidobacteriales	BMI	1035	1.07	[1.01, 1.14]	1.36 × 10 − 2	FALSE	FALSE	FALSE
	WHRadjBMI	565	1.06	[0.99, 1.13]	0.12	FALSE	FALSE	FALSE
Family Bifidobacteriaceae	BMI	1035	1.07	[1.01, 1.14]	1.36 × 10 − 2	FALSE	FALSE	FALSE
	WHRadjBMI	565	1.06	[0.99, 1.13]	0.12	FALSE	FALSE	FALSE

Pleiotropy was detected using the MR-Egger intercept (i.e., P_Egger-intercept < 0.05). Heterogeneity was detected using Cochran’s Q statistics (i.e., *P* < 0.05). The association was considered to be driven by a single SNP if the association was no longer nominally significant (i.e., *P* > 0.05) when that SNP was removed from the analysis.

*BMI* body mass index, *WHR* waist-hip ratio, *OR_SD* odds ratio per standard deviation, *CI* confidence interval, *N_SNP* number of IVs used in the MR analysis.

**Fig. 2:**
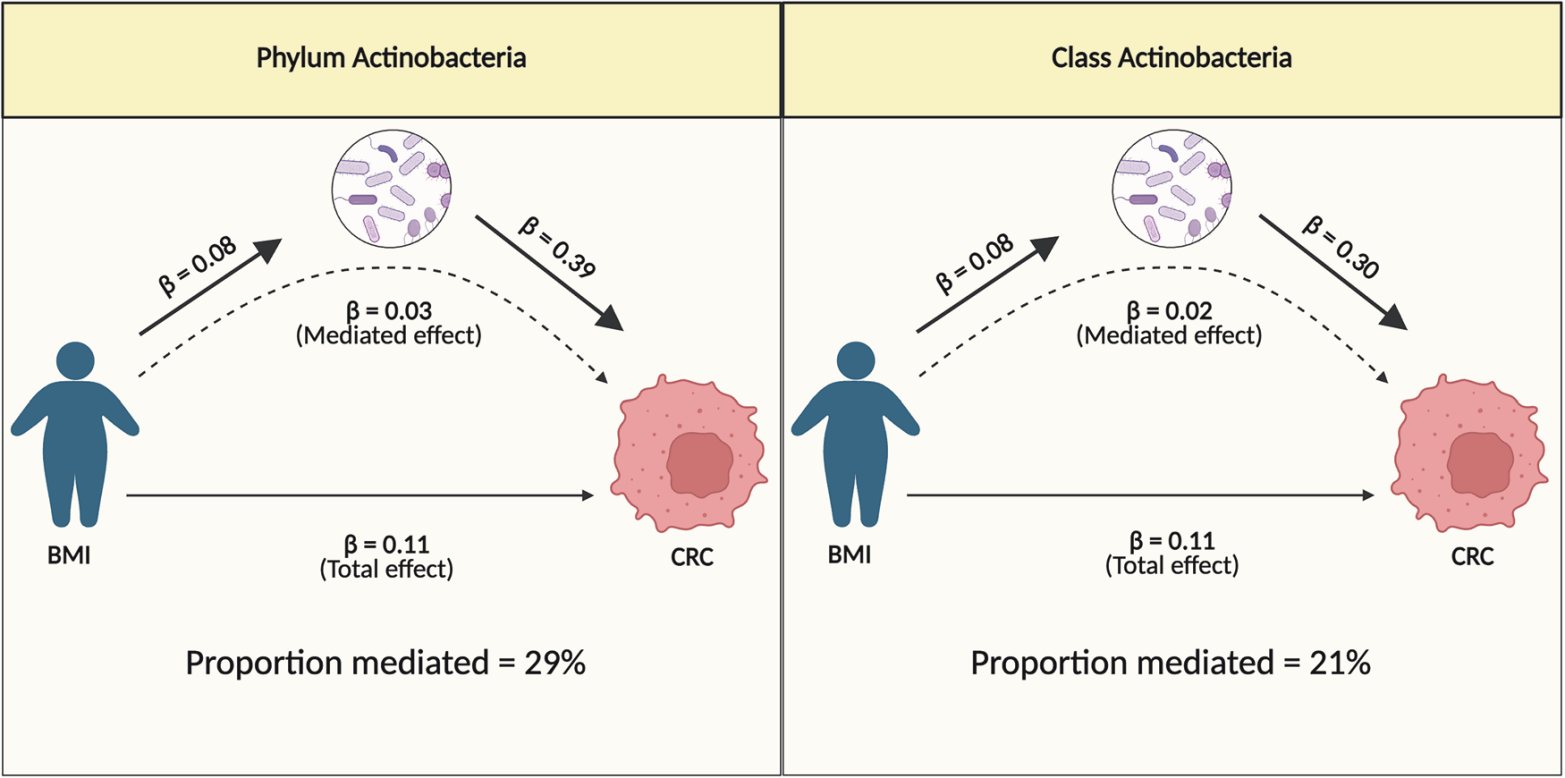
Mediation of BMI-driven CRC risk by Actinobacteria taxa. Effect sizes and mediated proportions were calculated according to Fig. 1. BMI body mass index, CRC colorectal cancer.

We next estimated the proportion of the effect of BMI on CRC risk mediated by the Actinobacteria taxa, using mediation analysis (Fig. 2). Firstly, the total effect of BMI on CRC risk was recalculated with the overlapping IVs excluded (see Methods) (OR_SD_: 1.12; 95% CI: [1.06, 1.17]). Then, the effect of BMI on phylum Actinobacteria (OR_SD_: 1.08; 95% CI: [1.03, 1.14]) and class Actinobacteria (OR_SD_: 1.08; 95% CI: [1.02, 1.14]) was recalculated with the overlapping IVs removed. Finally, the mediated effect size was estimated as the product of the genetically predicted effect of a per S.D. increase in BMI on the abundance of Actinobacteria and the genetically predicted effect of a per S.D. increase in the abundance of Actinobacteria on CRC risk. Both phylum (OR_SD_: 1.03; 95% CI: [1.01, 1.06]) and class Actinobacteria (OR_SD_: 1.02; 95% CI: [1.00, 1.04]) showed a significant mediated effect. Taking the ratio of the mediated and total effects gives the proportion of the total effect of BMI on CRC risk mediated through phylum (proportion mediated: 29%; 95% CI: [8,50]) and class Actinobacteria (proportion mediated: 21%; 95% CI: [4,37]).

No pleiotropic associations were observed with alcohol consumption, diet, or physical activity [25]. However, three of four IVs for Actinobacteria were associated with lipid metabolites (S16 Table), consistent with possible metabolic mediation rather than horizontal pleiotropy [28, 29].

## Discussion

Our study, using 2S-MR and mediation analysis, has utilised one of the largest GWAS of the microbiome to date [30] and found potential associations linking Actinobacteria taxa with obesity-driven cancer risk. We also report associations between the gut microbiome and CRC risk that require further study to determine if the relationships are robust.

Although our analyses support a mediative role of the microbiome on cancer risk, a number of methodological considerations are pertinent. While the majority of participants in the MiBioGen consortium were of European ancestry, approximately a quarter of participants were of other ancestries. This could lead to false positives or insufficient power to detect true associations, due to population heterogeneity [30]. Associations between phylum/class Actinobacteria and CRC risk have previously been reported [31] in 2S-MR analysis using the non-population-stratified parent study [32] of the CRC GWAS data employed here. In that study, the direction and size of the effects when using 254,791 individuals with European or east Asian ancestry were consistent with the effect found here, using only European-ancestry individuals. The replication of the effects in both homogeneous and heterogeneous populations ensures that the detected associations were not driven by population stratification arising from the non-European ancestry individuals in the MiBioGen GWAS data.

In contrast to our analysis, nominal BMI associations with breast, ovarian and prostate cancers have been reported [33], but none reached the conservative threshold applied here to reduce type 1 error in the mediation analysis. Furthermore, a recent study found opposite directions of effect for the genera Tyzzerella3 and Ruminococcus torques group with CRC risk [34]. Inspection of the analysis shows that this appears to be ascribable to a methodological error in their analysis pipeline. Based on the analysis of 57,889 CRC cases, Li et al. [35] reported a negative association between CRC risk and Tyzzerella3 using reverse MR. We observed no significant association in our larger CRC dataset (Table S11). In a meta-analysis of fecal shotgun metagenomic studies of colorectal cancer [36], genus Tyzzerella and species Ruminococcus torques showed significantly reduced abundance in CRC cases. This is in line with the findings reported here, although care must be taken when interpreting the results of Wirbel et al., given that CRC is likely to disrupt the gut microbiome homeostasis.

Altering the gut microbiome through diet or supplements may provide a method to reduce CRC risk in individuals with obesity. However, class and phylum Actinobacteria are highly diverse taxa and understanding the effect on CRC risk of individual genera or species within these taxa is required before the gut microbiome is an actionable target. This is underscored by the fact that Bifidobacteria, a member of the Actinobacteria phylum, is known to increase anti-tumour immune response [37], contrary to our findings. This suggests that the relationship between Actinobacteria and CRC risk is complex and may reflect taxon-level heterogeneity or context-dependent effects. To identify the specific microbe(s) driving the association with BMI and CRC risk, better stratified GWAS of microbes within the Actinobacteria phylum are required.

In addition, the microbiome has been shown to change with age [38]; however, the MiBioGen GWAS is not age stratified and contains samples from both adults and children. The lack of IVs for most microbial taxa is also indicative of the necessity for further microbial GWAS with larger sample sizes and more precise taxonomic classification [30]. For example, observational studies have reported a negative association between obesity and family Christensenellaceae [39, 40]. However, we could not test for an association between Christensenellaceae and CRC risk due to the absence of suitable instruments in the microbiome GWAS.

Nevertheless, we performed a speculative investigation into the pathway through which Actinobacteria may mediate obesity-driven CRC risk. IVs for both phylum and class Actinobacteria are known variants for lactase nonpersistence [41, 42], with one of the variants (rs182549-C) reported to be associated with an increase in colorectal cancer risk [43]. Lactase nonpersistence is associated with a lower dairy intake [42], and although there is conflicting evidence for an association between lower dairy intake and increased risk of obesity [44, 45], these findings suggest that diet, as shaped by lactase genotype, may influence the microbiome-cancer relationship. Future observational studies of obesity, diet, and colorectal cancer risk would ascertain whether dairy intake is a mediator of obesity-driven colorectal cancer risk.

In conclusion, our study found that class and phylum Actinobacteria may mediate up to half of the effect of obesity on CRC risk, with a number of other taxa potentially influencing CRC risk. Microbiome GWAS with better stratification and sample sizes may allow for the identification of the specific causal microbes within the Actinobacteria phylum and the development of treatments to mitigate obesity-driven CRC risk.

## Supplementary information

Supplementary Tables 1–16

## Data Availability

Instrumental variables are given in Tables S3–5. Summary GWAS BMI/WHRadjBMI data are available from https://portals.broadinstitute.org/collaboration/giant/index.php/GIANT_consortium_data_files. Summary GWAS cancer data are available from: https://bcac.ccge.medschl.cam.ac.uk/bcacdata/ (breast cancer); http://practical.icr.ac.uk/blog/?page_id=8088 (prostate cancer); GWAS Catalogue ID: GCST004481 (ovarian cancer); GWAS Catalogue ID: GCST006465 (endometrial cancer); GWAS Catalogue ID: GCST004748 (lung cancer); direct communication with consortia (renal and esophageal cancers); - phs001415.v1.p1, phs001315.v1.p1, phs001078.v1.p1, phs001903.v1.p1, phs001856.v1.p1 and phs001045.v1.p1 (US based studies) and GWAS Catalogue ID: GCST90129505 (European based studies) colorectal cancer. FinnGen data can be accessed by following the instructions at https://www.finngen.fi/en/access_results. Summary GWAS statistics for the microbiome are available at https://mibiogen.gcc.rug.nl/menu/main/home/. All code packages used in the analysis have been referenced at the appropriate points in the Methods section.
